# Evaluation of the Oxidative Stress Response of Aging Yeast Cells in Response to Internalization of Fluorescent Nanodiamond Biosensors

**DOI:** 10.3390/nano10020372

**Published:** 2020-02-20

**Authors:** Kiran J. van der Laan, Aryan Morita, Felipe P. Perona-Martinez, Romana Schirhagl

**Affiliations:** Department of Biomedical Engineering, University Medical Center Groningen, University of Groningen, Antonius Deusinglaan 1, 9713 AV Groningen, The Netherlands; kiranvanderlaan@gmail.com (K.J.v.d.L.); drg.armorita@gmail.com (A.M.); felipeperona@gmail.com (F.P.P.-M.)

**Keywords:** fluorescent nanodiamond, nitrogen-vacancy (NV) center, biocompatibility, oxidative stress, aging

## Abstract

Fluorescent nanodiamonds (FNDs) are proposed to be used as free radical biosensors, as they function as magnetic sensors, changing their optical properties depending on their magnetic surroundings. Free radicals are produced during natural cell metabolism, but when the natural balance is disturbed, they are also associated with diseases and aging. Sensitive methods to detect free radicals are challenging, due to their high reactivity and transiency, providing the need for new biosensors such as FNDs. Here we have studied in detail the stress response of an aging model system, yeast cells, upon FND internalization to assess whether one can safely use this biosensor in the desired model. This was done by measuring metabolic activity, the activity of genes involved in different steps and the locations of the oxidative stress defense systems and general free radical activity. Only minimal, transient FND-related stress effects were observed, highlighting excellent biocompatibility in the long term. This is a crucial milestone towards the applicability of FNDs as biosensors in free radical research.

## 1. Introduction

Fluorescent nanodiamonds (FNDs) are gaining more attention for the possibilities they offer in biomedical research. When using FNDs as free radical detectors in living cells, an abundance of knowledge on the ins and outs of oxidative stress and metabolism will be revealed. Free radicals are small molecules with a free electron in their outer orbit; a great part of them are derived from oxygen, and therefore some are also called reactive oxygen species (ROS). An unbound electron makes a molecule highly reactive towards other molecules, disrupting these in order to obtain a neutral charge. ROS are known for their two-sided effects. On the one hand, they are important in redox biology and signaling inside metabolically active cells, while on the other hand, when their normal balanced presence is disturbed, they can play an important role in oxidative stress and damage. Therefore, they are involved in many different diseases, such as cancers, cardiovascular diseases and viral and bacterial infections, as well as in aging [1,2].

The unpaired electron in radicals functions as a target for diamond magnetometry. The fluorescent nanodiamonds (FNDs) used for this act as magnetic sensors as they respond to the magnetic fields produced by the free electron spins. The FNDs harbor a defect in the diamond structure, in which two carbon atoms in the lattice are substituted by a nitrogen atom (N) and an adjacent vacancy (V). The so-called NV centers can be read out optically, since the fluorescence signal of the diamonds changes depending on the magnetic surroundings [3]. Diamond magnetometry with NV centers has already been successfully used to measure single electrons [4], for example, and even nuclear spins [5], magnetic vortices [6], a hard drive [7,8], nanoparticles [9] and other diamond defects [10].

Since the natural occurrence of ROS in the human body, there are cellular antioxidant defense systems present in the cells. These are activated when there is a change in ROS levels, in an attempt of the cell to deal with the disturbed balance. The primary antioxidant defense system in yeast comprises enzymes, which function as catalysts in processes to convert/metabolize the oxidants. Additionally, there are also non-enzymatic antioxidants that function as neutralizing agents by scavenging, of which the most abundant is glutathione [11,12].

In order to further develop fluorescent nanodiamonds to be used as a magnetic sensor to detect these ROS, and to enable the study for aging at a molecular level, FNDs were introduced in yeast cells. Yeast is a well-known cellular model to study aging. In earlier studies, the proliferation capacity and the aging curves of the cells were shown to remain unaffected after the introduction of FNDs [13]. However, there are other ways of assessing the cell viability and evaluating the response of cells. Prior to further investigation of the actual role of ROS in aging mechanisms, the oxidative response of yeast cells to FND internalization should be examined with state of the art methods, as was done previously for mammalian cells [14]. This is crucial to discriminate a possible FND-related effect from an age-related effect, which will be the subject of future studies.

Here we have firstly measured the metabolic activity. Next, we evaluated stress response in more detail, by evaluating the relative gene expression of oxidative stress genes that are active in different steps and locations of the oxidative stress defense system. Lastly, we have also measured the general presence of free radicals. With these experiments, we aim to characterize the oxidative stress response of yeast cells to the FND sensor internalization itself.

## 2. Materials and Methods 

### 2.1. Nanodiamonds

In this study, fluorescent nanodiamonds (FNDs) with an average hydrodynamic diameter of 70 nm (Adamas Nano, Raleigh, NC, USA, https://www.adamasnano.com/) were used. These are produced by grinding high-pressure high-temperature diamonds and irradiation with three MeV electrons to 5 × 10^19^ e/cm^2^ fluence to raise the number of NV centers to an average of 300 centers per diamond (determined by electron paramagnetic resonance by the manufacturer) [15]. The surface chemistry of the FNDs is oxygen-terminated, as a result of an acid treatment by the manufacturer. The uptake of FNDs into yeast cells was obtained by causing a temporary chemical permeabilization of the cell wall, using a transformation mix with 66.6% (w/v PEG4000) and 1 M lithium acetate (protocol as described by Hemelaar and van der Laan et al. 2017 [16]).

### 2.2. Yeast Strain and Cell Handling

A *Saccharomyces cerevisiae* strain from the yeast GFP Clone Collection from ThermoScientific (Waltham, MA, USA) was used [17], namely the BY4741 Hxt6-GFP strain. In this strain, a glucose transporter (HXT6) was fused with green fluorescent protein (GFP). These yeast cells were kept in synthetic dextrose (SD) complete medium (2% glucose, 6.9 g/L yeast nitrogen base without amino acids, 0.79 g/L dropout complete mix), in order to stabilize the genetic modification.

During multi-day experiments, cells were kept in a non-dividing state to represent so-called chronological aging. To reach this state, the medium was replaced by water after growing an overnight culture in 12–20 h towards the end of logarithmic growth (optical density of 0.6–1.0 A_600_) at 30 °C on a shaking platform (200 rpm). Cells in this phase were diluted to a final concentration of 1 × 10^8^ cells/mL. Cells were washed two times and spun down by centrifuging (2400 g, 6 and 12 min), and were then resuspended in 20 mL sterile H_2_O and kept at 30 °C with shaking.

### 2.3. Sample Conditions

The following cell conditions were used in each experiment: the negative control sample consists of cells without an uptake procedure, and without FNDs, the chemical transformation sample (CT) was subjected to the uptake protocol without the addition of FNDs, and the chemical transformation + FNDs sample (CT + FNDs) was subjected to both the uptake protocol and the FNDs. These conditions were applied to be able to discriminate effects of either the CT or the FNDs. The positive control was different depending on the experiment: cells were either subjected to boiling temperatures (to affect metabolic activity) or to H_2_O_2_ (to induce oxidative stress). After the different treatments, cells were kept in a non-dividing state in water. Later measuring time-points are therefore long-term measurements during chronological aging.

### 2.4. Metabolic Activity

To assess the metabolic activity after the uptake protocol and the addition of FNDs, an adjusted MTT assay protocol for yeast cells was performed [18]. Cells were incubated with a 10% MTT solution (3-(4,5-dimethylthizaol-2-yl)-2,5-diphenyltetrazoliumbromide, Sigma Aldrich, Zwijndrecht, The Netherlands, https://www.sigmaaldrich.com/) in the dark for two hours, and subsequently spun down at 17,000g for 10 min. Afterwards, the cells were dissolved using 100% dimethyl sulfoxide (DMSO) [19,20], prior to the detection of the absorption using a FLUOstar Omega Microplate Reader (BMG Labtech, De Meern, The Netherlands, https://www.bmglabtech.com/) at 540 nm. After correction for the background signal, the obtained signals are given as a percentage of the negative control. A metabolic activity of between 80% and 120% was considered as normal.

### 2.5. qPCR of Oxidative Stress Genes

Intracellular mRNA transcription levels of role-players in the oxidative defense system were evaluated by qPCR. The following genes were selected:

- Enzymatic response: catalase (CTT1, CTA1), superoxide dismutase (SOD1, SOD2) and thioredoxin.
- Non-enzymatic antioxidant scavenger that was tested: glutathione (GSH1, GSH2).
- Three reference genes were selected: ALG9, TAF10 and TFC1 [21].

Cell pellets for qPCR were shock-frozen using liquid nitrogen and stored at −80 °C until further analysis. For RNA isolation, the samples were dissolved in RNase free water and crushed by using N_2_ grinding. Using an RNA lysis buffer with 1% 1 M dithiothreitol, the powder was taken, and next the InviTrap Spin Universal RNA Mini Kit (Stratec molecular, GmbH, Berlin, Germany, https://www.invitek-molecular.com/) was used to isolate the RNA. The obtained RNA was converted into copy DNA using the iScript Advanced cDNA Synthesis Kit (Bio-Rad, Hercules, CA, USA, https://www.bio-rad.com/), and the concentration of obtained cDNA was measured by the NanoDrop ND-1000 UV/Vis Spectrophotometer (Nanodrop technologies, Wilmington, DE, USA). The CAS-1200 Robotic Liquid Handling System (QIAGEN, Corbett Robotics, Germantown, MD, USA) was used to set up the PCR plate. The actual real-time polymerase chain reaction (PCR), the amplification of cDNA, was performed using the SsoAdvanced Universal SYBR Green Supermix (Bio-Rad, Hercules, CA, USA) and the PrimePCR primers for the tested genes (Biorad, Hercules, CA, USA). As advised by the manufacturer, the following PCR program was run at the CFX384 Touch Real-Time PCR detection system (Bio-Rad, Hercules, CA, USA): 95 °C for 30 s, then 40 cycles at 95 °C for 10 s and 60 °C for 30 s, and finally an increase from 65 °C to 95 °C (with steps of 0.5 °C, each for five seconds).

Relative quantification was calculated using the standard curve-based method for relative real-time PCR data analysis. Data analysis was performed based on the starting quantity (SQ) values resulting from the dilution series of a cDNA mix applied in each qPCR run. SQ values were first referenced against the average of the three reference genes, to account for the general activity of the samples [21]. Next, the SQ values were normalized against the control sample with untreated cells; thus, the relative mRNA expression levels are depicted as fold change compared to the control. All samples were measured in duplicates and repeated in 3–4 independent qPCR runs. Data are shown as the average of the number of repetitions of qPCR runs (*n* = 3 for the H_2_O_2_ conditions, *n* = 4 for the experimental conditions). For each condition and time point, comparisons were made by comparing against the negative control, and significance was tested by performing an unpaired t-test with Welch’s correction using GraphPad Prism 6 software (San Diego, CA, USA) (significance depicted above the bars). Additionally, a comparison was made for each gene between the CT sample and the CT + FND sample at each time point.

### 2.6. Fluorescent Marker: DCDFA

To get a rough impression on the overall ROS activity in the cells, DCFDA (2′,7′-dichlorodihydrofluorescein diacetate) was used. This is an indirect measure for the total ROS production inside a cell, since it is only detected after entering the cell and after oxidation by ROS. DCFDA is deacetylated and oxidized to 2′,7′-dichlorodihydrofluorescein (DCF), which is fluorescent. A DCFDA protocol adjusted for yeast was used [22]. In order to account for the cells that die in response to H_2_O_2_ treatment (positive control), the detected DCFDA signal for a specific condition was referenced to their corresponding proliferation counts; given values are thus the product of the DCFDA activity and the proliferative ability.

After subjecting the cells to the chemical transformation and/or the diamonds, cells were incubated with 25 µM DCFDA (ThermoFisher, dissolved in DMSO) in a 96-well plate for two hours at 30 °C. Afterwards, the fluorescence was detected using a FLUOstar Omega Microplate Reader (BMG Labtech, De Meern, The Netherlands) at an excitation/emission of 485/520 nm. After correction for the background signal, the obtained signals are normalized against the untreated cells. Data are thus presented as a percentage of the control.

### 2.7. Statistical Data Analysis

All presented data were analyzed using GraphPad Prism 6, for both the preparation of visualizations and the performance of statistical tests. When comparing two conditions, statistical differences were tested by performing a multiple comparison t-test using the Holm-Sidak method at a significance level of 0.05.

## 3. Results

In previous publications [13,16], we have shown that cells survived the presence of FND. In this paper we take biocompatibility one step further. Here we investigate the cell’s response to the FNDs in greater depth by evaluating non-fatal changes in cellular activity. For these experiments, aging cell populations were prepared, and after treatment the cells were kept in water to allow chronological aging. Long-term measurements at later time points were thus performed with chronologically aged samples.

### 3.1. No FND-Induced Reduction in Metabolic Activity

**Figure 1:**
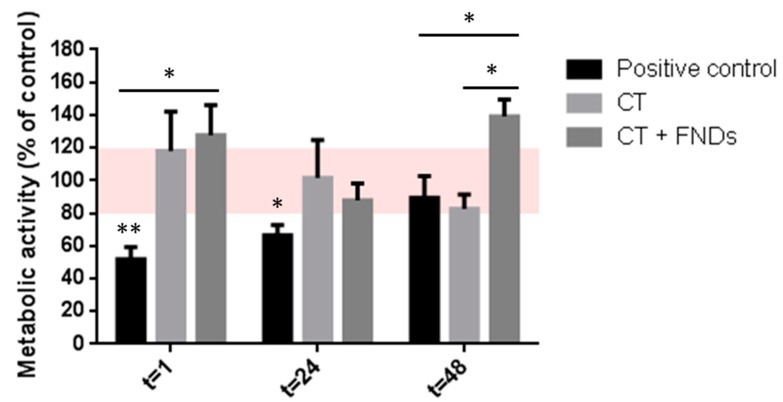
Metabolic activity of cells after uptake protocol and/or addition of FNDs. In the positive control, cells were incubated in boiling water for 20 min; the CT sample has been subjected to the chemical transformation only; and the CT + FND sample has been subjected to both the chemical transformation and the FNDs. The activity is given as a percentage of the negative control; the viability between 80% and 120% (red area) is considered to be unaffected. Error bars show the standard error of the mean. Significance is tested against the negative control (* *p* < 0.05, ** *p* < 0.01).

At first, the metabolic activity of the cells was measured directly after the introduction of the FNDs, as well as both 24 h and 48 h later (Figure 1). A decrease of the metabolic activity was observed in the positive control, incubated in boiling water, indicating a decreased viability as expected. More importantly, this affected viability was not observed in the cells that were treated with the uptake protocol (CT) and fluorescent nanodiamonds (FNDs); this means that the cells are not damaged in the same way as by the physical stress of the high temperature. Interestingly, whereas the cells of the positive control seemed to recover their activity towards 100%, the cells treated with CT and FNDs appeared to vary a bit more over time. The samples treated with either CT or CT + FNDs were not significantly different from untreated cells at any of the time points. Directly after treatment, the metabolic activity seems a bit higher for the treated cells. After 24 h, the metabolic activity seems to be restored back to 100%, and 48 h later we see that the sample with CT + FNDs shows an elevation in metabolic activity. Directly after and in the first day after treating the cells, there was no difference between the cells only treated with the chemical transformation and the cells that had additionally been subjected to FNDs. Interestingly, 48 h after the treatment, we did find a significant increase in the metabolic activity of the sample with FNDs.

### 3.2. Transient FND-Induced Changes in Oxidative Stress Transcriptome

Next to measuring the metabolic activity in general, we have specifically analyzed the mRNA expression of genes that are involved in the antioxidant defense system of the cells. To assess whether the presence of FNDs provoked a response from this antioxidant defense system, the expression levels of several players in this defense system have been analyzed. The defense system can be divided into an enzymatic and a non-enzymatic part. The expression levels of the key role players in the enzymatic response that were tested in this paper are catalase, superoxide dismutase and the key player in the thioredoxin antioxidant system. One of the important players of the non-enzymatic, scavenging response of the defense system is the antioxidant glutathione. Here we have tested the expression of GSH1 and GSH2, which are involved in two different steps of GSH synthesis.

Additionally, we have measured the expression levels of YAP1, which is a transcription factor involved in the oxidative stress during chronological aging.

**Figure 2:**
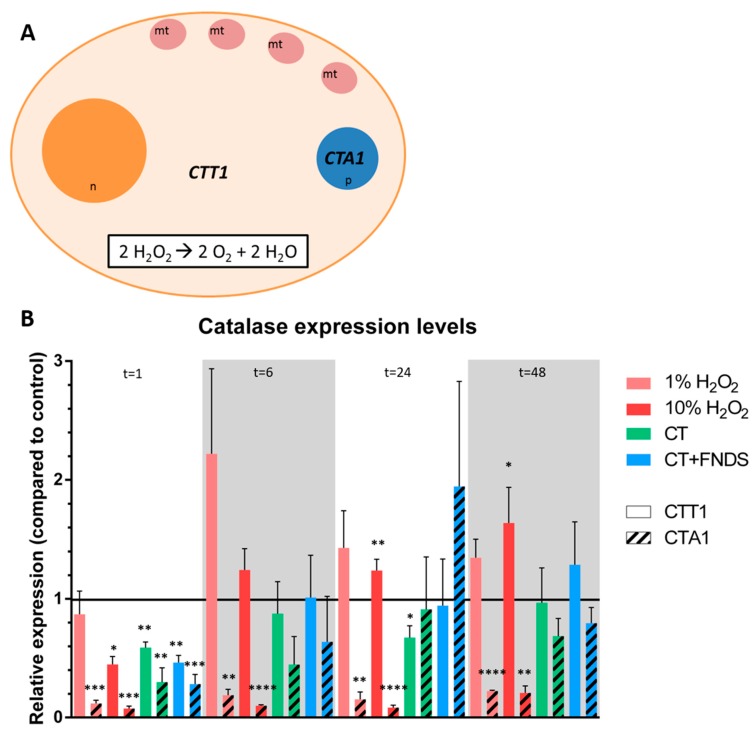
(**A**) Schematic representation of the cell showing the locations of catalase activity, including the reaction that is catalyzed by the catalase enzyme at both the cytoplasm (CTT1) and the peroxisomes (CTA1). (n = nucleus, mt = mitochondria, p = peroxisome); (**B**) Relative expression of catalase at different time-points: catalase T which is present in the cytoplasm (CTT1, filled bars) and peroxisomal catalase A (CTA1, striped bars). The colors represent different conditions, measured at 1/6/24/48 h, respectively. Values are averages out of three independent qPCR runs that were performed in duplicates. Error bars show the standard error of the mean. Significance was tested against the control (* *p* ≤ 0.05, ** *p* ≤ 0.01, *** *p* ≤ 0.001, **** *p* ≤ 0.0001).

The enzymatic activity of catalase effectuates the conversion of hydrogen peroxide into oxygen and water (Figure 2A). In yeast, the production of catalase can occur in the cytoplasm (CTT1) or in the peroxisomes (CTA1). For both catalases, a decrease in expression levels was observed directly after treating the cells (Figure 2B). However, the decrease in catalase expression appeared to resolve over time, and was back at the expression levels of untreated cells after 48 h.

Besides, the peroxisomal CTA1 expression appeared to be reduced by H_2_O_2_ treatment in the chronologically aging yeast at all the measured time points, in contrast to the cytoplasmic CTT1 expression that was only increased at higher H_2_O_2_ concentrations in the longer term.

**Figure 3:**
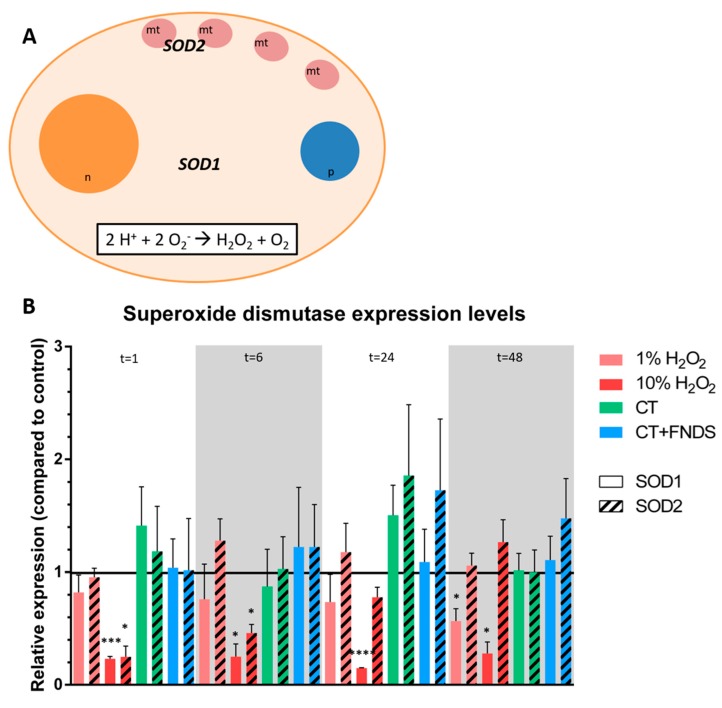
(**A**) Schematic representation of the cell showing the locations of superoxide dismutase activity, including the reaction that is catalyzed by the superoxide dismutase enzyme at both the cytoplasm (SOD1) and the mitochondria (SOD2). (n = nucleus, mt = mitochondria, p = peroxisome); (**B)** Relative expression of superoxide dismutase at different time-points: superoxide dismutase [Cu-Zn] which is present in the cytoplasm (SOD1, filled bars) and mitochondrial superoxide dismutase [Mn] (SOD1, striped bars). The colors represent different conditions, measured at 1/6/24/48 h, respectively. Values are averages out of 3–4 independent qPCR runs that were performed in duplicates. Error bars show the standard error of the mean. Significance was tested against the control (* *p* ≤ 0.05, ** *p* ≤ 0.01, *** *p* ≤ 0.001, **** *p* ≤ 0.0001).

The other tested enzyme, superoxide dismutase, catalyzes the conversion of superoxide in less damaged cells using copper/zinc, in the case of cytoplasmic SOD1, or manganese for the mitochondrial SOD2 (Figure 3A). In contrast to catalase, there were no changes in SOD expression measured after treating the cells with chemical transformation and/or FNDs at any of the measuring time points (Figure 3B). After 24 h, a tentative increase seemed to occur, but was not significant, and disappeared again at 48 h.

Additionally, the expression of the key player of the thioredoxin system was evaluated. No differential expression levels were detected for thioredoxin reductase as a result of treatment with FNDs responsible for the reduction of the oxidized antioxidant thioredoxin (Figure S1).

**Figure 4:**
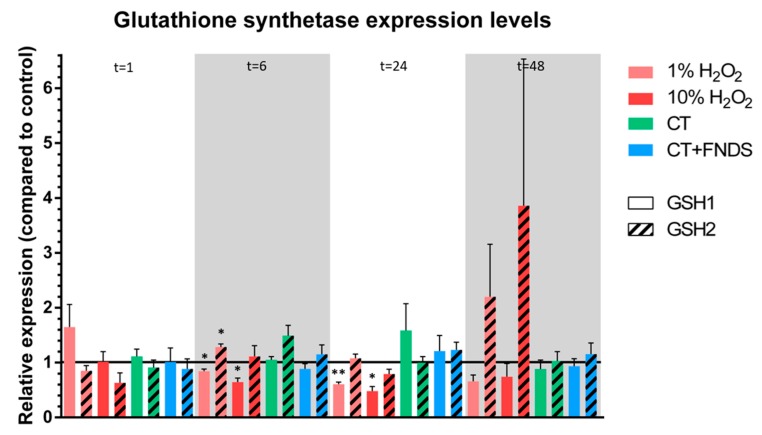
Relative expression of glutathione synthetases, both the enzyme that is involved in the first step of glutathione synthesis (GSH1, filled bars) and the enzyme that is involved in the ATP-dependent step of glutathione synthesis (GSH2, striped bars). The colors represent different conditions, measured at 1/6/24/48 h, respectively. Values are averages out of 3–4 independent qPCR runs that were performed in duplicates. Error bars show the standard error of the mean. Significance was tested against the control (* *p* ≤ 0.05, ** *p* ≤ 0.01).

To evaluate the non-enzymatic defense system, it was chosen to analyze the synthetase expression levels of the most abundant cellular antioxidant: glutathione (GSH). GSH1 catalyzes the first step in GSH biosynthesis, while GSH2 catalyzed the adenosine triphosphate (ATP)-dependent synthesis of glutathione. In general, the GSH expression levels of chronological aging yeast cells did not respond strongly to H_2_O_2_-induced oxidative stress, except from a non-significant increase in GSH2 expression after 48 h (Figure 4). Again, there were also no significant differences between the samples treated with the chemical transformation only and the ones additionally subjected to the FNDs.

**Figure 5:**
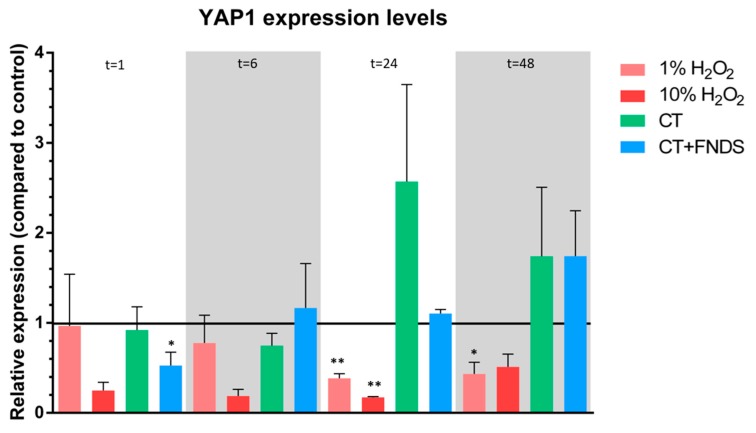
Relative expression of YAP1, the primary transcription factor involved in antioxidant response. The colors represent different conditions, measured at 1/6/24/48 h, respectively. Values are averages out of 3–4 independent qPCR runs that were performed in duplicates. Error bars show the standard error of the mean. Significance was tested against the control (* *p* ≤ 0.05, ** *p* ≤ 0.01).

Lastly, the relative expression levels of the transcription factor YAP1 were determined after treatment with chemical transformation and FNDs. YAP1 is a transcription factor involved in protective mechanisms in stress situations, more specifically by regulating the transcription of target genes encoding the yeast antioxidant response. During chronological aging, YAP1 was shown to be able to prevent the apoptosis-induced death of cells during chronological aging [23,24]. An increase in YAP1 expression levels seemed to occur at later time points, while a downregulation was only found for the cells treated with both the chemical transformation and the FNDs. Besides this, however, after up to 48 h no more significant aberrations of YAP1 expression were observed, along with an increasing chronological age (Figure 5).

Interestingly, the tested genes SOD and YAP1 have been shown before to be involved in chronological aging. SOD1 and SOD2 were shown to be involved in extending the survival lifespan of yeast [25], and a similar role was shown for YAP1. This gene is known to regulate genes encoding proteins involved in oxidant defense, and it has been shown to be able to improve survival during chronological aging by preventing the apoptosis-induced death of cells (the main cause for dying of cells during chronological aging) [23,24]. Here we have shown that FNDs, in the longer term, do not interfere with this YAP1-mediated oxidative stress defense during chronological aging. This corresponds to the earlier reporting of no significant differences in the survival of a chronological aging cell population after treatment with CT and FNDs [13].

### 3.3. Unaltered Total Free Radical Activity after Diamond Internalization

**Figure 6:**
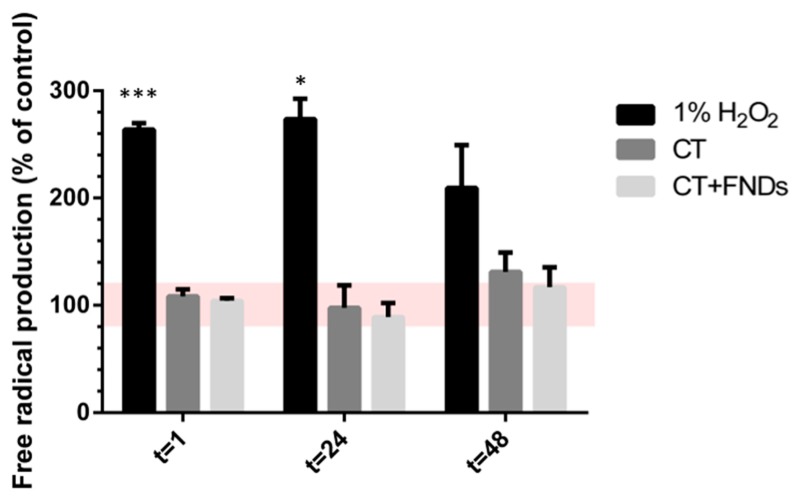
Total free radical production (measured by conversion of DCFDA to DCF). Yeast cells without a stimulant were used as a negative control to relate all values to. Values represent averages out of three biologically independent experiments, each performed in quadruplicate. Error bars represent standard errors of the mean. Significance was tested against the control (* *p* ≤ 0.05, *** *p* ≤ 0.001).

In addition to the genetic assessment of the oxidative stress response, the total free radical production was tested by a DCFDA assay. Directly after the treatment of cells with H_2_O_2_ to induce an oxidative stress response, a major increase in the total free radical production was observed (Figure 6). This effect slowly disappeared over time, indicating the restoration of the remaining chronologically aging cells. In chronological aging, the accumulation of free radicals is known to be involved in causing apoptosis, which is the cause of cell death during chronological aging. Accordingly, in the treated cells we see an overall increase in total free radical production over time (from 100% up to ~120%). Furthermore, at all three time points there was no difference in free radical production after either just the chemical transformation or both the chemical transformation and the addition of FNDs.

## 4. Discussion

### 4.1. Diamond Internalization Inaging Yeast Cells Does Not Provoke a Prolonged Oxidative Stress Response

Prior to the use of FNDs as free radical biosensors, here we have examined whether FNDs or their internalization had an effect on free radicals and oxidative stress themselves. After checking for changes in the general metabolic activity (MTT), here we have tested factors of both the enzymatic and nonenzymatic antidoxidant defense system of yeast cells. The primary antioxidant defense systems are induced upon oxidative stress, whenever the free radical balance is disturbed.

Although some differences were observed after treating the aging yeast cells with our nanodiamonds, these were mostly non-significant. Two interesting comparisons were made: location-dependent expression between members of the same gene cluster and differential expression in response to either only the chemical transformation or both the chemical transformation and the FNDs.

The location-dependent expression was investigated, as we have previously shown the preferred subcellular localization of FNDs close to membrane-enclosed structures (such as mitochondria and peroxisomes) [13]. We did not find differential expressions for catalases expressed in either the peroxisomes or the cytoplasm. For superoxide dismutase, there is a minimal increase in mitochondrial SOD2 expression as compared to its cytoplasmic expression. However, this was only seen at the later time points, and the upregulation was not significant. Notably, at these time points, the majority of the FNDs have already left the cells again, as we have shown before that the FND internalization was successful but transient.

Furthermore, the presence of FNDs in addition to the chemical transformation treatment did not have an additional effect. Within the first hour after the transformation and addition of FNDs, there was a differential expression for two of the tested genes (catalase and YAP1). At the next point in time, these effects were no longer significant, although since the next measurement was taken six hours after treatment, it remains unknown how long the effect stayed within these six hours. This is something that should be taken into account when continuing these experiments. It should also be noted, however, that this effect was not long-lasting, since the effects were no longer present after six hours. In a few conditions and time points, the chemical transformation seemed to have some effect, although the additional exposure to FNDs did not have an enhanced effect. This indicates that there is no significant effect of the FNDs on the oxidative response of the cells. Additionally, this actually shows that biological variance is a bigger factor in differential expression levels than our intervention (performing the chemical transformation and/or adding the FNDs).

### 4.2. The Many Faces of Cell Viability and the Challenges in Measuring Oxidative Stress Levels

Determining cell viability is a simple way to evaluate the stress condition of a cell population in unicellular organisms. Since the reduction of the growth rate is related to stress conditions, cell viability measurements are used to determine whether a stress response is activated [11]. The standard for cell viability determination is to count the number of colony-forming units, though this shows only one aspect of viability: the capacity to proliferate. Next to proliferation, one can also test the metabolic activity (e.g., by an MTT assay) or the membrane integrity (e.g., by a LIVE/DEAD assay) as measures for cell viability. These viability effects have been tested in response to nanodiamonds; FNDs were shown to have only minor effects on the proliferation capacity of yeast cells [13], and likewise only minor changes in functional effects were observed for several mammalian cell types [14,26,27,28], demonstrating the excellent biocompatibility of FNDs. When focusing more on detail on oxidative stress, there are different strategies to explore this. In this study, the level of oxidative stress was determined by analyzing both the relative mRNA expression of oxidative stress genes and by detecting the total free radical production using a fluorescent marker.

Changes in the oxidative stress transcriptome are used as a measure to evaluate to what extent the oxidative stress defense system is activated by our treatment (the chemical transformation and/or the FNDs). Whereas the mRNA analyses give an indication of the initial stress response and activity of certain genes, additional proteome analysis could give an accurate measure of the proteins that are present in these cells. This could be an interesting follow-up study, since the proteins are the molecules actually carrying out functions, to see whether any of the weak transcriptome changes actually result in a change of protein levels [11,29].

Another way to measure the activity of the antioxidant defense system is to use enzymatic activity assays, such as fluorometric catalase or GSH activity detection. These activity assays are based on the detection of fluorescent products, which means they are indirect measurements. Taking into account the highly reactive character of free radicals, this complicates the interpretation of results. This accounts somewhat for the total free radical activity measurement that was also used in this study. DCFDA is actually one of the most used assays for free radical activity, due to its simplicity and user-friendliness. While the assay is designed to measure the oxidation of DCFDA to the fluorescent DCF, it is questionable whether this oxidation reaction is induced only by cellular ROS. Moreover, this results in a lack of selectivity, as the probe reaction involves a multi-step process, allowing other species to be involved and preventing the assignment of the response to one reactive oxygen species in particular [30,31,32]. In general, this is one of the problems with using fluorescent probes for free radical measurements; the fluorescence intensity might be amplified/affected by any interactions with intermediate radicals.

Sensitive and reliable methods to detect free radicals are therefore still limited, due to their high reactivity, low baseline concentrations and short lifetimes. Using FNDs as biosensors would be a way to partly overcome these drawbacks, emphasizing the need for the further development of applicability for FNDs as free radical biosensors.

## 5. Conclusions

In order to validate if FNDs themselves influence the system we intend to measure, here we have investigated non-fatal changes in aging yeast cells in response to the internalization of our fluorescent nanodiamonds. First, there were no changes in metabolic activity up to two days after the treatment. Additionally, the presence of FNDs did not provoke a prolonged oxidative stress response in the aging yeast cells. While there was an effect for two of the genes at short notice, this differential expression did not last in the longer term. This is an important milestone that confirms the excellent biocompatibility of FNDs, and supports their usability in aging research as sensors in the search for the exact role of free radicals.

## Supplementary Materials

The following are available online at https://www.mdpi.com/2079-4991/10/2/372/s1, Figure S1: Relative expression of TRR1, key regulatory enzyme of the thioredoxin system.
