# Supplementary material for: Evaluation of the Oxidative Stress Response of Aging Yeast Cells in Response to Internalization of Fluorescent Nanodiamond Biosensors
Source: Nanomaterials (Basel). 2020 Feb 20;10(2):372. doi: 10.3390/nano10020372 (PMC7075316; doi:10.3390/nano10020372)
Supplement: Supplementary file 1 [file nanomaterials-10-00372-s001.pdf]

Supplementary

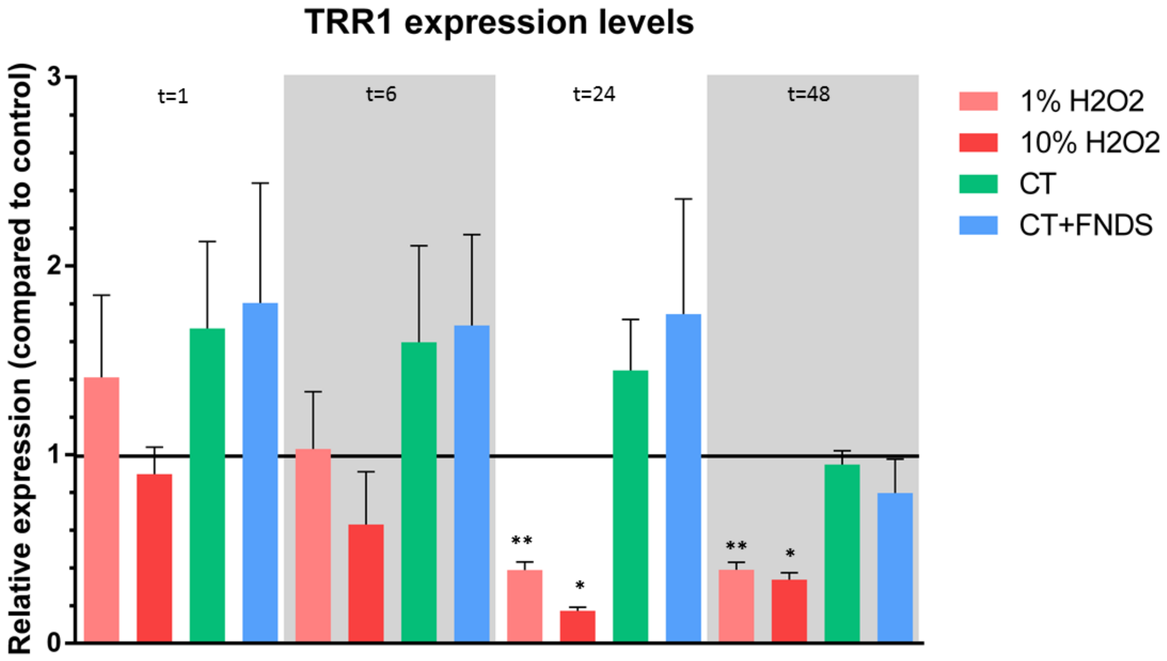

Figure S1. Relative expression of TRR1, key regulatory enzyme of the thioredoxin system. The colors represent different conditions, measured at 1/6/24/48 hours respectively. Error bars show the standard error of the mean. Significance tested against the control (*p≤0.05, **p≤0.01).
